# Whole genome sequence data on Ethiopian key sorghum landraces and founder lines

**DOI:** 10.1016/j.dib.2026.113197

**Published:** 2026-08-25

**Authors:** Meseret Wondifraw, Tokuma Legesse Guta, Arlyn J. Ackerman, Madhav Subedi, Firomsa Ayele Demisse, Mohamed Salah Hamid, Rebecca Cubitt, Jared L. Crain, Craig T. Beil, Moira J. Sheehan

**Affiliations:** a Breeding Insight, Cornell University, Ithaca, 14853, NY, USA; b Ethiopian Institute of Agricultural Research (EIAR), Melkassa Agricultural Research Center, PO Box 436, Adama, Ethiopia; c Breeding Insight, University of Florida, Gainesville, 32611, FL, USA; d Department of Plant Pathology, Kansas State University, Manhattan, 66506, KS, USA

**Keywords:** Sorghum, Ethiopia, Whole genome sequencing, Landraces, Founder lines, Agroecology, Adaptation, Photoperiodism, Genetic diversity

## Abstract

Sorghum (*Sorghum bicolor (L.)* Moench) is the fifth most important cereal globally. Its genetic diversity is key to improving yield stability, stress tolerance, and adaptation to different environments. Ethiopia is one of the centers for the crop’s origin, diversity, and use in both human food and livestock feed. However, genomic data on Ethiopian sorghum remain limited, especially for landraces preferred by local farmers. This dataset consists of whole-genome sequencing data for 188 Ethiopian sorghum accessions, including founder lines and important landraces from major agroecological zones. Sequencing was performed using the Complete Genomics DNBSEQ-T7 platform, generating high-coverage whole-genome data (20 × coverage). On average, each accession produced 64.7 million reads. Reads were aligned to the Sorghum bicolor NCBIv3 reference genome and variants called using GATK HaplotypeCaller with joint genotyping (GATK v4.6.1.0). Hard-filtering followed GATK best-practice thresholds (QD <2.0, FS> 60.0, MQ <40.0, MQRankSum <−12.5, ReadPosRankSum <−8.0), retaining biallelic SNPs with mean depth 10–50×, missingness ≤20%, and MAF ≥0.05, yielding 6095,752 high-confidence SNPs across 185 accessions. Both the raw FASTQ files and processed VCF files are publicly available to support studies of sorghum genetic diversity, population structure, selection, and the genetic basis of important traits.

Specifications Table

**Table:** Summary of dataset

Subject	Biology
Specific subject area	*Whole genome sequencing and variant discovery in sorghum*
Type of data	Raw, Analyzed, Filtered, processed
Data collection	Leaf tissue was sampled from 188 sorghum accessions and silica-dried prior to DNA extraction. Genomic DNA was extracted using the E-Z Plant DNA DS kit and quantified using Promega Quantiflour with a plate reader. Sequencing libraries were prepared using the NEBNext UltraExpress FS DNA Library Prep Kit and sequenced on the Complete Genomics DNBSEQ-T7 platform to generate 150-bp paired-end reads targeting ∼20 × genome coverage per accession. Reads were quality-checked, trimmed, and aligned to the *Sorghum bicolor* NCBIv3 reference genome for genome-wide variant discovery.
Data source location	Plant material was collected and sampled at the Melkassa Agricultural Research Center, Ethiopian Institute of Agricultural Research (EIAR), Adama, Ethiopia. DNA extraction and initial quality assessment were conducted at Cornell University, Ithaca, NY, USA. Sequencing was performed by Solis Agrosciences (St. Louis, MO, USA), and bioinformatic processing and quality control were carried out by Breeding Insight at Cornell University, Ithaca, NY, USA.
Data accessibility	Repository name: NCBI Sequence Read Archive (SRA); Zenodo Data identification number: SRA: PRJNA1428287, Zenodo: DOI: 10.5281/zenodo.21679027 Direct URL to data: SAR**:**https://www.ncbi.nlm.nih.gov/bioproject/PRJNA1428287 Zenedo: https://doi.org/10.5281/zenodo.21679027
Related research article	None

## 1 Value of the Data

• The dataset contains 188 Ethiopian sorghum accessions, including important landraces and founder lines from the country's main agroecological zones. Since Ethiopia is a center of origin and diversity for sorghum, these accessions capture a wide range of genetic variation encompassing locally adapted varieties and farmer-preferred types, including dual-use accessions.
• Whole-genome sequences were generated at 20x coverage, providing even, reliable coverage for high-confidence variant ascertainment. This dataset supports detailed analysis of genetic diversity and population structure, helping to identify important genomic regions and alleles for sorghum breeding. The dataset is a strong foundation for discovering SNPs, haplotypes, and other genetic variants relevant to sorghum improvement, supporting genome-wide association studies (GWAS), marker-assisted selection, and genomic selection. This dataset fills an important gap in sorghum genomic resources, both geographically and ecologically. It adds high-quality genomic data from Ethiopian germplasm to global diversity panels, enabling studies of regional adaptation, ancestral diversity, and stress-resilience-related traits for crop improvement.
• The geo-referenced collection sites provided for each accession offer a basis for genome-environment association (GEA) analyses linking genetic variation to local climate conditions across Ethiopia's agroecological zones, an approach increasingly used in crop landscape genomics. This can further support genomic prediction models for traits related to drought and heat tolerance when paired with phenotypic data.

## 2 Background

Sorghum (*Sorghum bicolor (L.) Moench*) is an annual diploid grass (2n = 2x = 20) belonging to the family Poaceae, subfamily Panicoideae. Sorghum originated in Africa, particularly in Sudan and Ethiopia, which remain important centers of diversity for both cultivated and wild forms [1]. Sorghum is a C₄ cereal characterized by efficient water and nutrient use, high photosynthetic capacity, and strong tolerance to drought and heat [2,3]. These traits make it well-suited to dryland farming systems and underscore its importance as a food, feed, and bioenergy crop for >500 million people worldwide [4]. In East Africa, sorghum is often cultivated as a dual-purpose crop, with many smallholders using the grain for food, the stalks for construction, and the leaves for animal feed [5]. Sorghum ranks as the fifth most important cereal crop globally, with approximately 57 million metric tonnes produced in 2023 [6]. The United States is a major producer, while Africa accounts for nearly 40% of global production, with Nigeria and Ethiopia among the region's leading producers [6].

Ethiopian sorghum is one of the most diverse groups within *Sorghum bicolor*. It includes four of the five main cultivated races, including durra, caudatum, guinea, and bicolor, as well as numerous intermediate forms distributed across heterogeneous agroecological zones [7,8]. This diversity is also reflected in flowering-time regulation, with some accessions exhibiting photoperiod-insensitive flowering [9], while others show strong photoperiod sensitivity [10,11]. These variations reflect adaptation to diverse rainfall patterns, seasonal length, and growing conditions across Ethiopia [12], and provide genetic variation for studying flowering-time regulation and environmental adaptation.

Several genotyping projects have advanced sorghum research using Ethiopian germplasm, including the ICRISAT mini-core collection [13], Ethiopian Core Collection [14], USDA-NPGS Ethiopian subset [11], and the G3 Ethiopian germplasm panel [15]. These datasets were generated using reduced-representation sequencing approaches such as genotyping-by-sequencing and DArTseq, typically producing 30,000–100,000 SNPs but with uneven genome coverage and high levels of missing data from sample to sample, which limit the ability to discover genome-wide variation and to accurately represent Ethiopia’s agroecological diversity.

The current dataset provides whole-genome sequencing (WGS) data for 188 Ethiopian sorghum accessions, including landraces from major production zones and founder lines prioritized by the Ethiopian Institute for Agricultural Research (EIAR) breeding program as sources of favorable alleles for crop improvement. The objectives are to: (1) capture the breadth of genetic variation in Ethiopian germplasm; (2) generate a comprehensive genomic dataset for variant discovery, population structure, and selection analyses; and (3) expand genomic resources through development of a targeted amplicon marker panel to support sorghum improvement.

## 3 Data Description

### 3.1 Germplasm and data availability

**Fig. 1:**
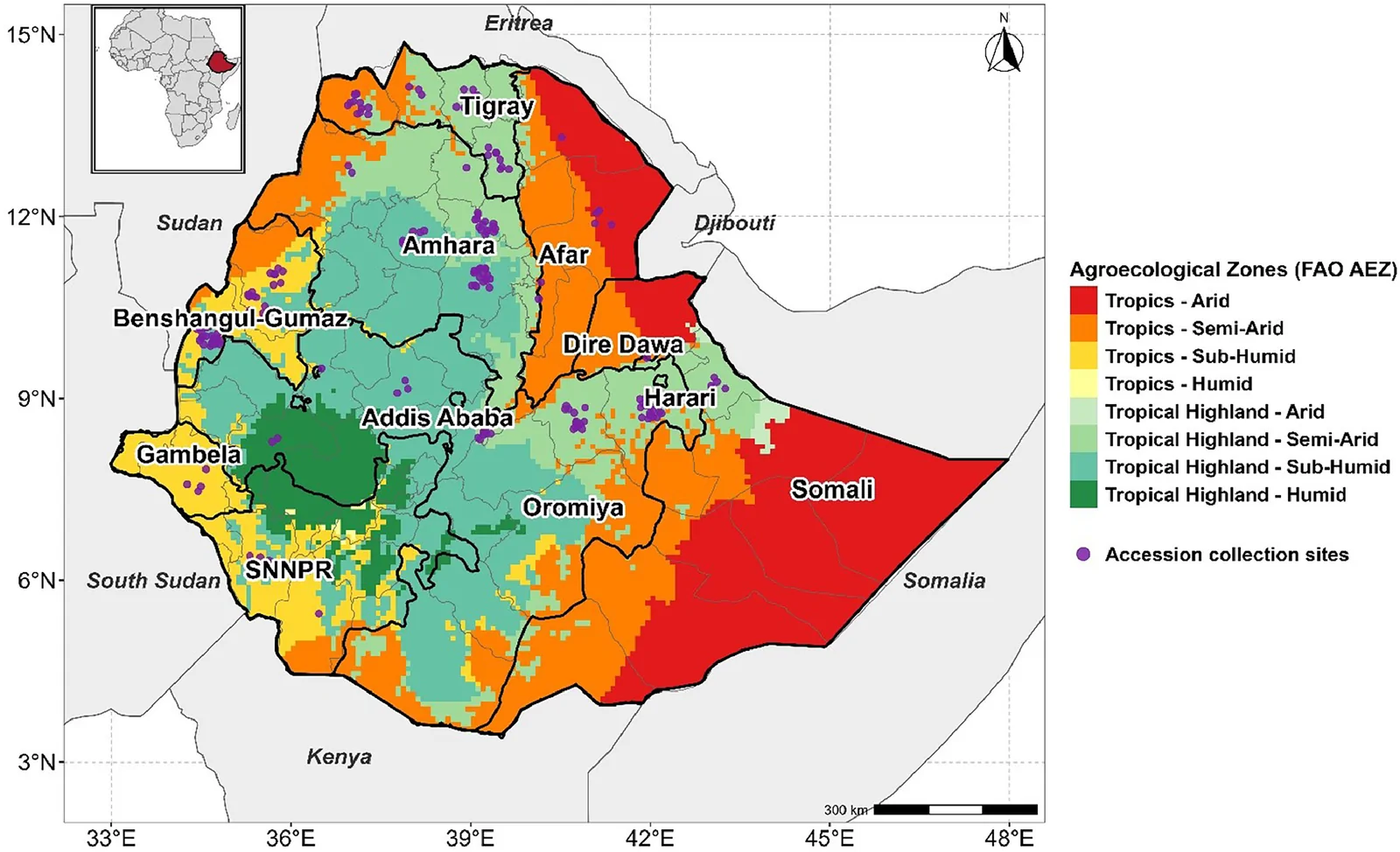
Geographic distribution of 188 Ethiopian sorghum germplasm accessions across major agroecological zones (FAO AEZ classification) and administrative regions**.** Purple points indicate accession collection sites. The inset map shows Ethiopia's location within Africa.

The dataset includes whole-genome sequencing data from 188 Ethiopian sorghum accessions. These samples come from major sorghum-growing regions across Ethiopia and represent the main agroecological zones where the crop is grown. The sampling covers arid, semi-arid, sub-humid, and humid areas, as well as lowland, intermediate, and highland environments with different temperatures, rainfall, and growing seasons (Fig. 1).

The dataset includes two primary groups: 77 founder lines and 111 key landrace accessions. The founder lines were prioritized by the national breeding program at the Ethiopian Institute of Agricultural Research (EIAR) and include released varieties, farmer-preferred cultivars, and selected accessions identified as sources of favorable alleles for genetic improvement. The remaining 111 accessions comprise key landraces maintained and cataloged by the program, representing locally adapted germplasm originating from diverse production regions.

The complete list of 188 *Sorghum bicolor* accessions, along with associated metadata, is provided in Supplementary Table 1. Raw FASTQ files for all 188 accessions have been deposited in the National Center for Biotechnology Information (NCBI) Sequence Read Archive (SRA) under BioProject accession PRJNA1428287. Genomic variant calls (VCF format) were generated by aligning reads to the *Sorghum bicolor* reference genome BTx623 (NCBI assembly GCF_000003195.3). Variant files were produced for the 185 samples that passed FastQC filtering, and the processed VCF files have been deposited in the Zenodo repository along with the accession metadata (https://doi.org/10.5281/zenodo.21679027), which include georeferencing and associated climatic information for each accession derived from administrative-level collection records and CHELSA-bioclim data. Researchers are encouraged to use this dataset for population genomics, diversity analysis, and breeding applications.

### 3.2 Genetic diversity of the accessions

**Fig. 2:**
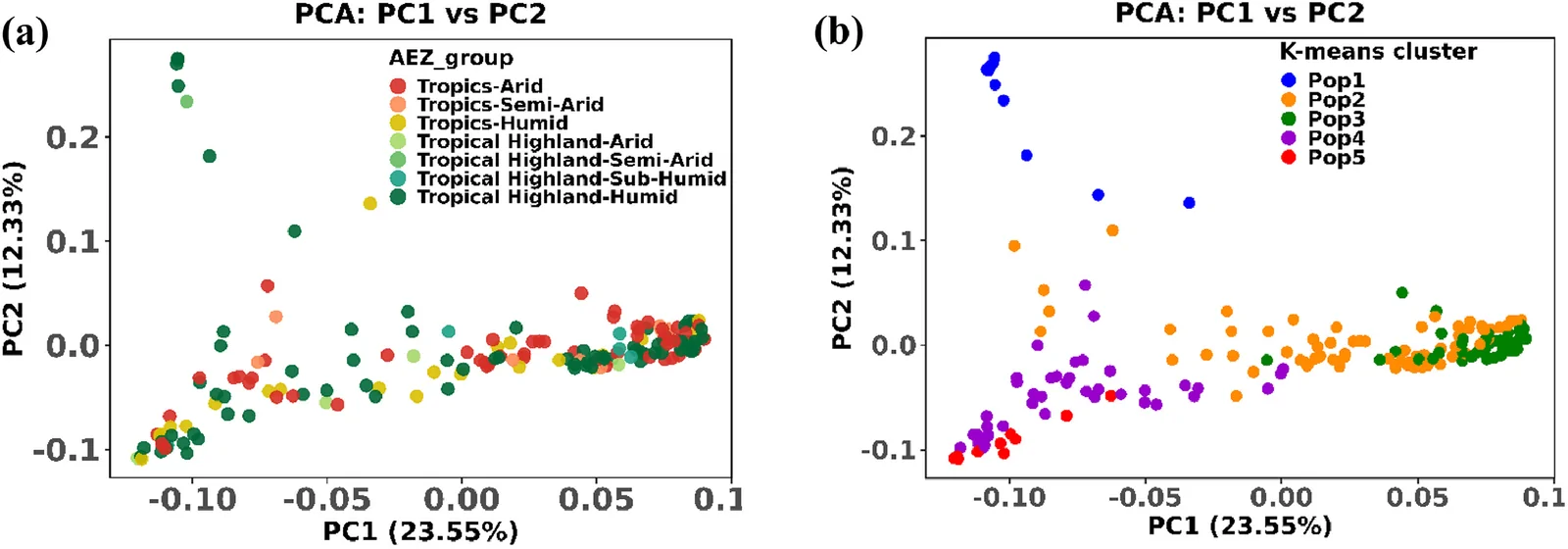
Principal component analysis (PCA) of 185 Ethiopian sorghum founder lines and landrace accessions based on genome-wide SNP markers. (a) Distribution of accessions in the principal component space colored by agroecological zone (AEZ). (b) The same PCA projection, colored by k-means cluster assignment (k = 5).

**Fig. 3:**
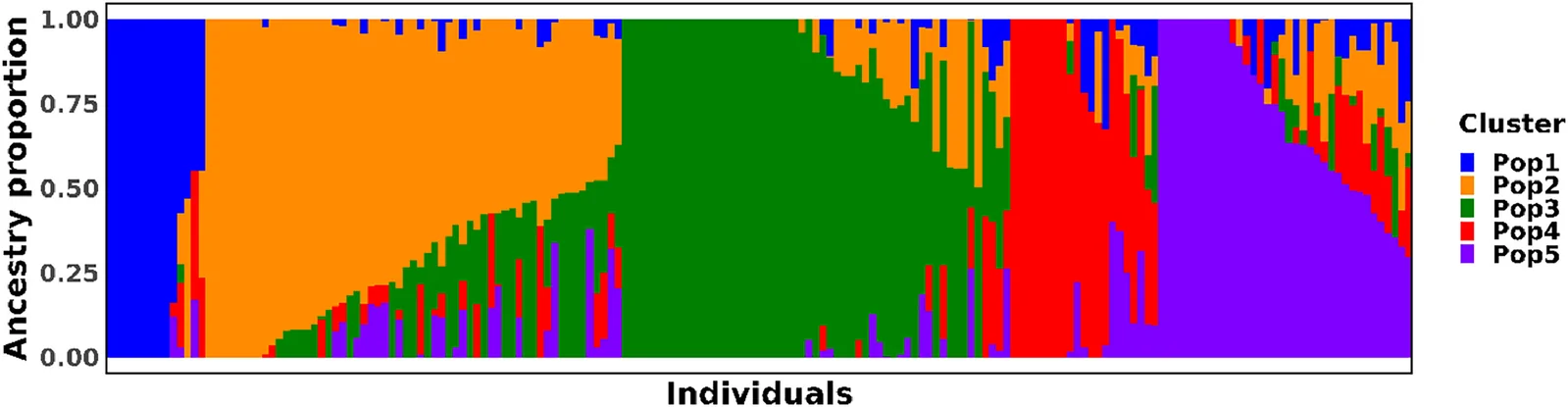
ADMIXTURE bar plot showing the ancestral proportions of 185 Ethiopian sorghum founder lines and landrace accessions at K = 5. Each vertical bar represents an individual accession, and colors indicate the proportion of ancestry assigned to each genetic component.

The germplasm panel showed substantial genome-wide genetic variation among accessions. The distribution of individuals across the principal component space revealed broad diversity, with some accessions forming tighter groupings while others occupying intermediate positions along the genetic gradient (Fig. 2a). When individuals are colored according to their dominant ADMIXTURE ancestry component, the distribution of accessions in the PCA space aligns with the five ancestry components identified by ADMIXTURE in this panel (Fig. 2b). The ADMIXTURE bar plot further shows the ancestry composition of each accession, with some individuals dominated by a single ancestry component and others displaying varying degrees of admixture (Fig. 3). These patterns highlight the diverse genetic backgrounds represented within the panel.

## 4 Experimental Design, Materials, and Methods

### 4.1 Tissue sampling

Leaf tissue sampling was conducted for 188 sorghum accessions, comprising 77 founder lines and 111 key landraces. Although sorghum is predominantly self-pollinating, limited natural outcrossing can occur [16,17], and many landrace accessions are bulked remnant seed from the prior season, and some outcrossing is expected. To capture any within-accession heterogeneity, six seeds per accession were planted in seedling trays and grown under shade cloth at the Melkassa Agricultural Research Center, Ethiopia (8°24′ N, 39°21′ E). When seedlings were one month old, one leaf disc was collected from each plant using sterile punches, and the six samples were pooled to form a composite tissue sample representing the accession. Samples were arranged in two 96-well plates, with two wells on each plate left empty as negative controls. Leaf tissues were desiccated over indicator silica gel in sealed containers to preserve DNA quality, according to the silica-drying protocol developed by the USDA–ARS Eastern Regional Small Grains Genotyping Laboratory (Raleigh, NC; 2016). The dried samples were shipped to Cornell University in April 2025 for DNA extraction.

### 4.2 DNA extraction, library preparation, and whole genome sequencing

DNA extraction was performed at the Genomics Facility of the Biotechnology Resource Center (RRID: SCR_021727) at Cornell University using the E-Z Plant DNA DS kit (https://omegabiotek.com/) and quantified using Promega Quantifluor and a plate reader. DNA concentrations ranged from 0.1 to 23.5 ng/µL. Most samples exhibited good quality and quantity of DNA suitable for whole-genome sequencing, while two samples exhibited low DNA yield and fragment integrity. Extracted DNA was sent to Solis Agrosciences (St. Louis, MO, USA) for library construction and sequencing. Sequencing libraries were generated using the NEBNext UltraExpress FS DNA Library Prep Kit (New England Biolabs) according to the manufacturer's standard protocol. The libraries were sequenced on the Complete Genomics DNBSEQ-T7 (https://www.completegenomics.com/) platform to produce 150-bp paired-end reads, targeting approximately 20 × coverage per accession.

### 4.3 Bioinformatic processing

Of the 188 DNA samples submitted, three did not meet the minimum sequencing depth requirement to pass quality control at Solis Agrisciences (St. Louis, MO, USA). The remaining 185 accessions produced high-quality data, averaging 64.7 million paired-end reads (2 × 150 bp) per accession and achieving a mean genome coverage of 27.3x relative to the *Sorghum bicolor* reference genome (Sorghum_bicolor_NCBIv3). Sequencing quality was evaluated by Breeding Insight (RRID:SCR_026645) using FastQC (v0.12.1) and summarized with MultiQC for consistency across samples. Adapter sequences and low-quality bases (Q < 20) were trimmed using Cutadapt (v5.1), and reads shorter than 30 bp were removed. Filtered reads were aligned to the *Sorghum bicolor* NCBIv3 reference genome using BWA-MEM (v0.7.18) for coverage and mapping evaluation, and alignment files were processed and evaluated using SAMtools (v1.21) and Picard (v3.3.0). After excluding three low-coverage samples, the remaining datasets showed coverage ranging from 9.1x to 30.3x (mean 22.2x). Summary statistics of sequencing depth per accession for the 185 samples that passed quality control are presented in Supplementary Table 2.

**Fig. 4:**
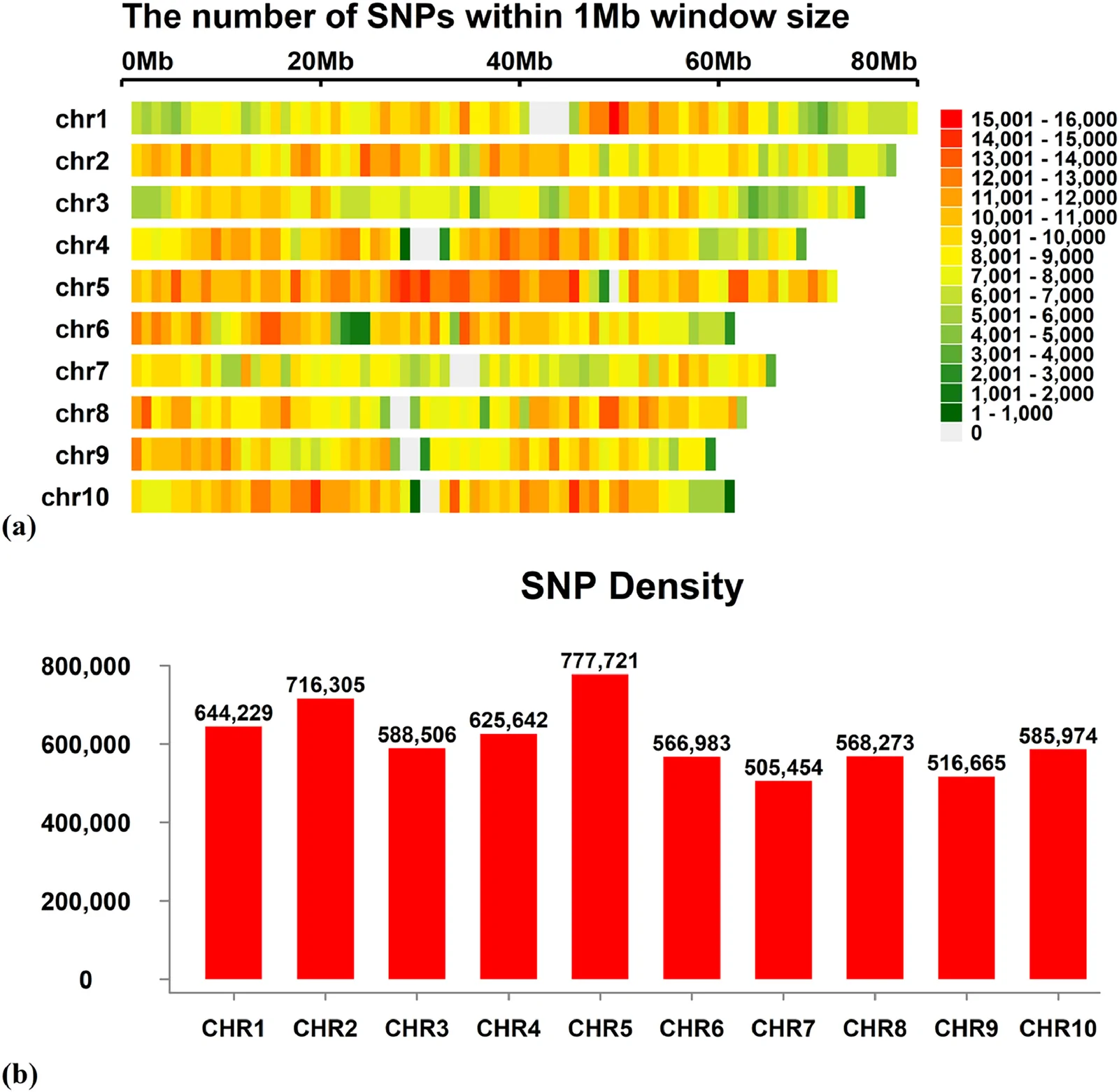
Genome-wide distribution of the 6095,752 filtered high-confidence SNPs identified across the 10 sorghum chromosomes in 185 Ethiopian sorghum accessions. **(**a**)** SNP density per 1 Mb genomic window, colored by marker count (grey indicates windows with zero SNPs). **(**b**)** Total number of filtered SNPs identified per chromosome.

Variants were called for each accession using GATK HaplotypeCaller (v4.6.1.0) in GVCF mode, and individual GVCF files were combined using GATK CombineGVCFs (v4.6.1.0) prior to joint genotyping with GATK GenotypeGVCFs (v4.6.1.0). Jointly genotyped variants were filtered using GATK (v4.6.1.0) recommended SNP hard-filtering thresholds to retain high-confidence sites. Variants with low quality relative to depth (QD < 2.0), strong strand bias (FS > 60.0), low mapping quality (MQ < 40.0), or evidence of mapping and positional bias (MQRankSum < −12.5; ReadPosRankSum < −8.0) were excluded. Multiallelic sites were excluded to retain only biallelic SNPs. Additional filters were applied to remove sites with insufficient or excessively high coverage (mean DP 10–50), high missingness (>20%), or low allele frequency (MAF < 0.05). The final dataset comprised 6095,752 high-confidence SNPs across 185 accessions (Fig. 4).

Population structure was assessed by principal component analysis (PCA) and ADMIXTURE. To reduce the influence of linkage disequilibrium on ancestry estimates, SNPs were pruned using *PLINK –indep-pairwise* 50 5 0.2, retaining 832,224 of the 6095,752 high-confidence SNPs. PCA was performed on this pruned marker set using PLINK, and ancestry proportions were estimated using ADMIXTURE across K = 2–10, with the optimal K selected by cross-validation error.

## Limitations

This dataset consists of whole-genome sequencing and genotype data only; it does not include phenotypic measurements. Users wishing to conduct trait-association studies (e.g., GWAS) will need to integrate this dataset with their own phenotypic data. Accessions were sampled at a single collection time point, so the dataset does not capture temporal or seasonal variation in agroecological conditions.

## Ethics Statement

The authors confirm that this study complies with the ethical standards required for publication in *Data in Brief*. The work does not involve human subjects, animal experiments, or data collected from social media platforms.

## Credit Author Statement

**Meseret Wondifraw:** Data curation, Formal analysis, Investigation, Methodology, Project administration, Software, Validation, Visualization, Writing- Original Draft Preparation, Writing- Review & Editing. **Tokuma Legesse Guta:** Conceptualization, Data curation, Funding acquisition, Methodology, Project administration, Supervision, Validation, Writing- Review & Editing. **Arlyn J. Ackerman,** Data curation, Project administration, Supervision, Writing- Review & Editing. **Madhav Subedi**: Methodology, Software, Writing- Review & Editing. **Firomsa Ayele Demisse:** Data curation, Investigation, Resources.**Mohamed Salah Hamid**: Investigation, Resources. **Rebecca Cubitt:** Resources, Writing- Review & Editing. **Jared L. Crain**: Funding acquisition**,** Writing- Review & Editing. **Craig Beil**: Conceptualization, Funding acquisition, Methodology, Project administration, Supervision, Writing- Review & Editing. **Moira J. Sheehan**: Conceptualization, Funding acquisition, Project administration, Supervision, Writing- Original Draft Preparation, Writing- Review & Editing

## Data Availability

NCBI SRAEthiopian Sorghum raw WGS SRA (Original data). ZenodoEthiopian Sorghum WGS (Original data).

## Appendix Supplementary materials

Image, application 1
